# Robotics in surgery: Current trends

**DOI:** 10.1016/j.amsu.2022.104375

**Published:** 2022-08-17

**Authors:** Hitesh Chopra, Atif Amin Baig, Simona Cavalu, Inderbir Singh, Talha Bin Emran

**Affiliations:** Chitkara College of Pharmacy, Chitkara University, Punjab, India; Unit of Biochemistry, Faculty of Medicine, Universiti Sultan Zainal Abidin, Kuala Terengganu, 20400, Malaysia; Faculty of Medicine and Pharmacy, University of Oradea, 10 P-ta 1 Decembrie, 410073, Oradea, Romania; Chitkara College of Pharmacy, Chitkara University, Punjab, India; Department of Pharmacy, BGC Trust University Bangladesh, Chittagong, 4381, Bangladesh; Department of Pharmacy, Faculty of Allied Health Sciences, Daffodil International University, Dhaka, 1207, Bangladesh

**Keywords:** Robotics, Laparoscope, Surgical navigation, Robots, FDA

Dear Editor,

When a procedure is called “robotic,” it does not indicate that a robot is really doing the work. Instead, it refers to surgical procedures in which doctors use robots to guide their actions. One or more robotic arms may be used in robotic surgical systems, which doctors can operate remotely and accurately from a console nearby. A laparoscope is attached to one of the robot arms. Small surgical tools may be carried in the other arms by the surgeons. The surgeon is able to see the tumor in three dimensions thanks to a computer screen. Each robotic arm is controlled by a joystick similar to that used in video games, which replicates the movements of the wrist and hand and provides dexterity.

In comparison to conventional laparoscopic or minimally invasive surgery, robotic devices are believed to have superior dexterity and range of motion. Surgeons may now operate on sections of the body that were previously inaccessible and get a better look at otherwise difficult-to-see areas. Minimally invasive operations often involve robots. Small incisions are used in these procedures, as the name suggests. Less discomfort, less bleeding, shorter hospital stays, and faster recovery periods are all common benefits of this approach.

The robotic helper was created to improve on already excellent minimally invasive surgery. In July 2000, the FDA approved the da Vinci Surgical System, the first surgical robot, for general minimally invasive surgery [1]. Robotic prostate removal (radical prostatectomy) was approved by the FDA in the following year. Cancer procedures including gynecological cancers were approved by the FDA in 2005. The Senhance System, a comparable robot, was approved by the FDA in 2017. Surgeons started employing robots for a broader variety of cancer operations as more hospitals purchased this $ 2 million equipment. Robotic assistants have been advertised as “where Star Trek meets Dr. Oz,” and many patients have sought them out as a result of seeing them in ads. Patients' desire for robotic procedures has fueled their wider acceptance, according to research. It's not certain whether utilizing a robot for cancer treatment is better than conventional methods, as the FDA cautioned in 2019.

Preoperative planning, surgical navigation, and surgical assistance may all be supported by a variety of computer-assisted surgical systems. An RAS device is a form of computer-aided surgical system that uses robots [2]. A range of surgical procedures may be performed with RAS devices, which let the surgeon to employ computer and software technology to control and move surgical tools via one or more small incisions in the patient's body (minimally invasive).

Some of the advantages of a RAS device may include its capacity to aid minimally invasive surgery and help with complicated tasks in restricted regions. In reality, the machine is not a robot since it cannot carry out surgery without direct human intervention. In the right hands and with the right training, robotic surgery may be a safe and effective tool for completing specific surgeries. It is the goal of the FDA to ensure that devices are safe and effective for their intended use [3]. Ensure manufacturers provide proper training for both new and seasoned users of RAS devices. Medical device training and education are not regulated or accredited by the FDA since they are not part of the agency's purview. It is up to companies like pharmaceutical companies, doctors' offices, and health care institutions to come up with and execute effective training programs. Specialty certification organizations and professional societies may also help to establish and fund training programs for its members' doctors of certain specialties. The certification status of their specialist doctors is likewise maintained by the specialty boards. The Hominis Surgical System, a revolutionary robotically-assisted surgical device (RASD), has been given the green light by the US Food and Drug Administration to go on sale in the United States. The Hominis Surgical System is designed for salpingo-oophorectomy in conjunction with benign hysterectomy (removal of the uterus for non-cancerous reasons) [4].

The Hominis Surgical System makes use of transvaginal (via the vagina) and laparoscopic (through a tiny incision in the belly) minimally invasive surgical equipment and a video camera to see the instruments within the patient during uterine removal. When compared to traditional laparoscopic surgery, the transvaginal method involves fewer abdominal incisions. Surgeons in the operating room use the Hominis Surgical System console to control the tools throughout the process. Before using the device, surgeons and operating room personnel must finish a rigorous training program developed and provided by the company.

Additional to testing for performance and engineering, the FDA examined 30 patients who had had transvaginal complete hysterectomy, salpingectomy, or salpingo-oophorectomy for benign diseases, all using the Hominis Surgical System. More than six out of ten patients had a variety of comorbidities, such as excessive cholesterol, osteoporosis, or high blood pressure, which varied in severity from patient to patient. Using the Hominis Surgical System, there were no conversions to an open or alternative laparoscopic surgical method in any of the 30 surgeries performed. Minor blood loss, urinary tract infection, and delayed healing of the top of the vagina (vaginal cuff) closure that is done as part of a hysterectomy were among the observed side effects.

The FDA has not approved any RAS systems for use in mastectomy patients, and there is insufficient data to support the use of RAS devices for breast cancer prevention or therapy. The surgical procedure for patients having a mastectomy is different when using RAS devices. It has not been proven how these changes would affect cancer prevention, overall survival, recurrence, and disease-free survival in the long run.

In terms of medical technology, robotic surgery is nothing new. Many hospitals are still reluctant to use robots for patients because of the high expenses, human resources, and lack of competence required.

The future of robotic surgery, on the other hand, shows a quick growth towards precise and least invasive versions of fundamental surgery. Post-op infections and other problems linked with typical open procedures will also be less common in patients who have had minimally invasive surgery. Robotic surgery is expected to be combined with other cutting-edge technology in the future. Surgeons' job may be made easier with the aid of artificial intelligence in the medical industry.

Though we may look to the future, robotic surgery will not be able to take the position of human doctors anytime soon. Robotic systems, in their most basic, are here to augment human abilities and improve post-operative results.

## Provenance and peer review

Not commissioned, internally peer-reviewed.

## Data statement

No specific data collected for the above manuscript.

## Ethical approval

Not applicable.

## Sources of funding

This study received no specific grant from any funding agency in the public, commercial, or not-for-profit sectors.

## Author contribution

**Hitesh Chopra:** Conceptualization, Data curation, Writing-Original draft preparation, Writing- Reviewing and Editing. **Atif Amin Baig:** Data curation, Writing-Original draft preparation, Writing- Reviewing and Editing. **Simona Cavalu:** Writing-Reviewing and Editing, Visualization. **Inderbir Singh:** Writing- Reviewing and Editing, Visualization, Supervision. **Talha Bin Emran:** Writing- Reviewing and Editing, Visualization, Supervision.

## Trail registry number

1. Name of the registry: Not applicable.

2. Unique Identifying number or registration ID: Not applicable.

3. Hyperlink to your specific registration (must be publicly accessible and will be checked): Not applicable.

## Guarantor

Talha Bin Emran, Ph.D., Associate Professor, Department of Pharmacy, BGC Trust University Bangladesh, Chittagong 4381, Bangladesh. T: +88-030-3356193, Fax: +88-031-2550224, Cell: +88-01819-942214. https://orcid.org/0000-0003-3188-2272. E-mail: talhabmb@bgctub.ac.bd.

## Consent

Not applicable.

## Declaration of competing interest

All authors report no conflicts of interest relevant to this article.

## References

[1] Yu J., Wang Y., Li Y., Li X., Li C., Shen J. (2014). The safety and effectiveness of Da Vinci surgical system compared with open surgery and laparoscopic surgery: a rapid assessment. J. Evid. Base Med..

[2] U.S. Food and Drug Administration (2019).

[3] Harvey B.E., Alpert S. (1997). Regulatory policy and computer assisted imaging technologies: is “virtual colonoscopy” an implied claim?. Gastrointest. Endosc..

[4] Lowenstein L., Mor O., Matanes E., Lauterbach R., Boulus S., Weiner Z., Baekelandt J. (2021). Robotic vaginal natural orifice transluminal endoscopic hysterectomy for benign indications. J. Minim. Invasive Gynecol..

